# Coping Strategies and Their Impact on Quality of Life and Physical Disability of People with Multiple Sclerosis

**DOI:** 10.3390/jcm10235607

**Published:** 2021-11-29

**Authors:** Silvia Cerea, Marta Ghisi, Marco Pitteri, Maddalena Guandalini, Lauren B. Strober, Simona Scozzari, Francesco Crescenzo, Massimiliano Calabrese

**Affiliations:** 1 Department of General Psychology, University of Padova, 35131 Padova, Italy; marta.ghisi@unipd.it (M.G.); scozzari.simona@gmail.com (S.S.); 2 Unit of Hospital Psychology, University-Hospital of Padova, 35131 Padova, Italy; 3 Department of Neuropsychology, National Hospital for Neurology and Neurosurgery, London WC1N 3BG, UK; 4 Department of Neurosciences, Biomedicine and Movement Sciences, University of Verona, 37134 Verona, Italy; maddalena.guandalini@univr.it (M.G.); francescocrescenzo89@gmail.com (F.C.); massimiliano.calabrese@univr.it (M.C.); 5 Kessler Foundation, West Orange, NJ 07936, USA; lstrober@kesslerfoundation.org; 6 Department of Physical Medicine & Rehabilitation, New Jersey Medical School, Rutgers, The State University of New Jersey, Newark, NJ 07103, USA; 7 Neurology Unit, Mater Salutis Hospital, AULSS 9, 37045 Verona, Italy

**Keywords:** coping, general distress, multiple sclerosis, health-related quality of life, physical disability

## Abstract

The aim of the study is to investigate the impact of coping strategies on Health-Related Quality of Life (HRQoL) and physical disability assessed with the Expanded Disability Status Scale (EDSS) of people with multiple sclerosis (pwMS). PwMS were asked to focus on “MS diagnosis” as the core stressor. One hundred eight pwMS completed the Coping Responses Inventory-Adult form (CRI-Adult), the Multiple Sclerosis Quality of Life-29 (MSQoL-29), and the Depression Anxiety Stress Scale-21 (DASS-21). Multiple regression analyses (first block: EDSS, disease duration, and DASS-21) revealed that physical MSQoL-29 was positively associated with Alternative Rewards and negatively with Resigned Acceptance of the CRI-Adult. The mental MSQoL-29 was positively associated with Problem-Solving and negatively with Emotional Discharge. The Expanded Disability Status Scale (EDSS; first block: disease duration and general distress) was negatively associated with Positive Reappraisal. The Analysis of covariance (ANCOVA) revealed that pwMS with lower physical disability showed higher scores in Positive Reappraisal and lower scores in Emotional Discharge than pwMS with a higher physical disability. Coping strategies can play a role on HRQoL and physical disability in pwMS above and beyond EDSS, disease duration, and general distress. Psychological interventions should be considered in pwMS since the time of diagnosis to promote engagement in adaptive coping strategies and contrast the maladaptive ones.

## 1. Introduction

Multiple Sclerosis (MS) is a chronic, inflammatory, and neurodegenerative autoimmune disorder of the human central nervous system (CNS) characterized by both white matter (WM) and gray matter (GM) damage [1] that leads to physical disability [2] and cognitive impairment [3]. Both physical disability and cognitive impairment can drive to psychological distress, which may result in substantial limitations of daily-life activities [4], and compromise Health-Related Quality of Life (HRQoL) of people with MS (pwMS) [5,6]. Adaptive coping is critical for adjusting to the demands of MS [7]. Coping is defined as the cognitive and behavioral efforts made to manage internal and/or external demands that challenge the person’s resources [8]. Individuals may develop habitual ways to manage stressors [9] or employ coping strategies based on the nature of the stressful situation [9]. In general, coping strategies are categorized as active/adaptive or avoidant/maladaptive [10]. Active coping involves adaptive strategies that best allow one to overcome stress and return to a healthy or desired state (e.g., strategies for acting). On the other hand, avoidant coping is comprised of less adaptive strategies (e.g., denial, behavioral and mental disengagement). In general, adaptive coping strategies predict better physical and mental health outcomes [11,12], whereas avoidant coping strategies are associated with negative physical and mental health outcomes [10,13] and poor Quality of Life (QoL; [14]).

Coping strategies may influence MS outcomes in different ways, including appropriate management of pain, adherence to treatment (e.g., physical therapy and medication), and promoting self-care activities (e.g., obtaining adequate rest). Therefore, in pwMS adaptive coping strategies can be helpful for their synergy with medical approaches to the disease in terms of early acute phase management and delay of disability progression.

A systematic review [15] has shown that pwMS employ emotional and avoidance coping strategies (e.g., denial, behavioral and mental disengagement) more frequently than problem-focused (adaptive) strategies (e.g., planning/activity), particularly in the early stages after the MS diagnosis. Such coping strategies [16] are associated with poor physical and psychological QoL [17,18,19], worse psychosocial adjustment [18,20], and greater depressive symptoms in pwMS [21,22,23].

Although coping strategies and their relationship with HRQoL have been widely assessed in pwMS, most studies have focused on habitual ways to manage stressors [24,25] but not on specific MS-related stressors (e.g., diagnosis, impact of early intensive treatment). Thus, few studies have focused on assessing coping strategies employed by pwMS when facing stressors specifically related to MS diagnosis. Moreover, previous studies assessing the relationship between coping and physical disability have shown conflicting results. For instance, several studies have shown that greater physical disability, as measured by the Expanded Disability Status Scale (EDSS; [26]), is associated with the employment of emotional and avoidance coping strategies [22,23,27,28]. Nevertheless, other studies did not find differences in coping strategies of pwMS in accordance with their level of physical disability [19,29,30,31]. Such findings might lead to ambiguity pertaining to what kind of coping strategies are mainly employed by pwMS likewise their level of physical disability, leaving some open questions that should be clarified to promote effective coping-based interventions for pwMS. Thus, exploring in greater depth the influence that coping may have on physical health outcomes is worthy of note [32].

The aim of this retrospective study was to unravel the impact of coping strategies employed by pwMS at the time of MS diagnosis on their physical disability and HRQoL by means of the self-report questionnaire Coping Response Inventory-Adult form (CRI-Adult; [33]). We asked pwMS to answer to the CRI-Adult items thinking specifically about their MS diagnosis as the core stressor. In accordance with previous studies [14,34,35], approach coping strategies are expected to predict better physical disability and HRQoL, whereas avoidance coping strategies are expected to have a negative impact on physical disability and HRQoL. To further explore the relationship between coping strategies and physical disability, we compared pwMS with normal or minimally disabling physical status to those with moderate or severe physical disability. We hypothesize that greater active coping is be adopted by the lower disability group, whereas more avoidance coping strategies are adopted by the higher disability group.

## 2. Materials and Methods

### 2.1. Participants

One hundred and eight pwMS (84 females) were included in the present study. Inclusion criteria were diagnosis of relapsing-remitting (RR) MS according to McDonald criteria [36], absence of concomitant neurologic or other pathologic health conditions, major psychiatric disorders, substance abuse, or severe cognitive impairment [37]. Secondary progressive (SP) course was defined by the occurrence of continuous disability accumulation over time, independently of relapses. Forty-seven pwMS were treated with dimethyl fumarate, 14 with fingolimod, 10 with interferon beta 1a, 8 with ocrelizumab, 4 with natalizumab, 4 with teriflunomide, 3 with glatiramer acetate, and 18 patients were untreated but monitored with clinical and radiological follow-up. At the time of testing, ninety-nine pwMS were still considered to be in the RR phase, while nine patients were in the SP phase. The mean age of participants was 38.09 years (SD = 10.63), whereas the mean education was 14.31 years (SD = 3.24). Pertaining to clinical variables, the mean disease duration (i.e., time from diagnosis) was 6.35 years (SD = 6.63) and the median EDSS was 1.5 (range: 0–6.5, irrespective of the last clinically evident attack).

### 2.2. Assessment

Coping Responses Inventory-Adult Form (CRI-Adult [33]; Italian version [38]): measure made up of 48 items assessing approach (logical analysis, positive reappraisal, guidance/support seeking, and problem-solving) and avoidance coping (cognitive avoidance, resigned acceptance, alternative rewards, and emotional discharge). The first two subscales of each domain (approach and avoidance) measure cognitive coping, whereas the second two subscales measure behavioral coping. Participants were asked to think specifically about how they cope with the diagnosis of MS when replying to the items of the questionnaire. Means and standard deviations obtained by pwMS on CRI-Adult are provided in the Appendix A.

Multiple Sclerosis Quality of Life-29 (MSQoL-29 [39]): a measure assessing HRQoL in pwMS. The higher the scores, the better the HRQoL. Physical Health Composite (PHC) and Mental Health Composite (MHC) can be computed. Means and standard deviations obtained by pwMS on MSQoL-29 are provided in the Appendix A.

Depression Anxiety Stress Scale-21 (DASS-21 [40]; Italian version [41]): 21-item scale assessing depression, anxiety, and stress, with higher scores indicating greater distress. Three subscale scores and the total score are computed [41]. To the current study, we focused only on the total score of the scale (i.e., general distress). Means and standard deviations obtained by pwMS on DASS-21 are provided in the Appendix A.

The Expanded Disability Status Scale (EDSS [26]) was administered by an experienced neurologist (M.C.) to investigate physical disability. The EDSS quantifies the physical disability level and ranges from 0 (no disability) to 10 (death) in 0.5 units increments that represent higher levels of disability. It is the most widely used instrument in clinical trials for the evaluation of the effectiveness of therapeutic interventions and in clinical practice for monitoring the disease activity and progression.

### 2.3. Procedure

Participants were recruited at the tertiary MS Center of Verona University Hospital. The study was proposed to each patient in conjunction with their routine neurological and neuropsychological evaluation as per clinical practice. Participants gave their written informed consent for participation and completed online self-report measures assessing coping strategies, HRQoL, and general distress within 14 days of the last visit. The study was approved by the Ethic Committee of Verona University.

### 2.4. Statistical Analyses

To investigate the relationship between coping strategies, physical disability, and HRQoL, Pearson’s correlation analyses were performed between coping strategies, HRQoL, general distress, disease duration, and level of physical disability. Based on correlational findings, three multiple regression models were performed. Pertaining to the role played by coping strategies on HRQoL, the MSQoL-29 physical (PHC) and mental health (MHC) composite scores were the dependent variables and the EDSS total score, disease duration, and DASS-21 total score were always included in the first block to control for physical disability status, disease duration, and general distress. Finally, coping strategies were entered in the second block. Pertaining to the role played by coping strategies on patients’ level of physical disability, EDSS was the dependent variable and, based on correlation findings, disease duration and DASS-21 were included in the first block; then, coping strategies were entered in the second block.

To examine the relationship between coping strategies and level of physical disability, we classified pwMS into two groups [26]: patients who presented with normal to minimal physical disability (EDSS ≤ 2; “normal status/minimal physical disability” group; *n* = 63) and patients who presented with moderate to severe physical disability (EDSS > 2; “moderate/severe physical disability” group; *n* = 45). This EDSS cut-off score was chosen to obtain a balanced distribution of the study population. Independent samples *t*-tests were performed to compare groups on demographic (i.e., age, and education) and MS-related (i.e., disease duration) variables. Given that groups differed with respect to disease duration, Analyses of Covariance (ANCOVA) were employed to compare the “normal status/minimal disability” group vs. the “moderate/severe physical disability” group on coping strategies considering disease duration as a covariate.

Finally, given that disease duration might have an impact on physical disability and HRQoL of pwMS [5,42], we divided the group by shorter (<5 years) or longer (≥5 years) disease duration since the time of diagnosis [43,44]. Given that the two groups differed about EDSS, ANCOVAs were employed to compare groups on coping strategies, considering EDSS as a covariate.

Statistical analyses were conducted by using IBM SPSS statistics (version 25, IBM Corp, Armonk, NY, USA, 2017).

## 3. Results

### 3.1. Correlational Findings

Correlational analyses revealed significant associations between coping strategies, physical and mental health (i.e., MSQoL-29), general distress, and clinical variables (i.e., disease duration and EDSS). Results are shown in Table 1.

### 3.2. Coping Strategies as Predictors of Physical Health

Based on the first-order correlations, problem-solving, cognitive avoidance, resigned acceptance, alternative rewards, and emotional discharge coping strategies were entered in the first regression model with physical HRQoL as the dependent variable. Disease duration, EDSS, and general distress were entered in the first step of the regression model to control for such variables. The overall model explained 51% of the variance (see Table 2) with disease duration, EDSS, and general distress accounting for 37.8% of the variance, F_(3102)_ = 20.65, *p* < 0.001, and coping strategies of resigned acceptance and alternative rewards explaining an additional 13.2% of the variance in the MSQoL-29 physical health (F change = 5.20; *p* < 0.001).

### 3.3. Coping Strategies as Predictors of Mental Health

Based on the correlational findings, problem-solving, cognitive avoidance, resigned acceptance, and emotional discharge subscales were included in the model, whereas disease duration, EDSS, and general distress were entered in the first step. The overall model explained 47.9% of the variance in the MSQoL-29 mental health with disease duration, EDSS, and general distress accounting for 39.3% of the variance, F_(3102)_ = 21.99, *p* < 0.001. The inclusion of coping strategies explained an additional 8.6% of the variance (F change = 4.06; *p* = 0.004; see Table 3). Specifically, results revealed that problem-solving and emotional discharge strategies were significantly associated with mental health.

### 3.4. Coping Strategies as Predictors of Physical Disability

Disease duration and DASS-21 total score were entered in the first step of the regression model, whereas coping strategies were entered in the second step. The overall model explained the 28.9% of the variance of the EDSS. Disease duration and general distress were significantly associated with EDSS, F_(2103)_ = 7.04, *p* = 0.001, explaining the 12% of the variance. The inclusion of coping strategies in the second step explained an additional 16.9% of the variance (F change = 7.93; *p* < 0.001; see Table 4). Controlling for disease duration and general distress, results revealed that positive reappraisal was significantly associated with EDSS.

### 3.5. Differences between “Normal Status/Minimal Physical Disability” (EDSS ≤ 2) and “Moderate/Severe Physical Disability” (EDSS > 2) Groups

Pertaining to disease duration (i.e., time from diagnosis), the “moderate/severe physical disability” group showed a longer disease duration than the “normal status/minimal disability” group. Pertaining to coping strategies, groups differed only with respect to positive reappraisal and emotional discharge coping strategies (*p* = 0.01 and *p* = 0.03, respectively): the “normal/minimal disability” group showed higher scores on the positive reappraisal subscale and lower scores on the emotional discharge subscale than the “moderate/severe disability” group. As a covariate, disease duration was not significant (respectively, *p* = 0.78; *p* = 0.31); however, disease duration emerged as a significant covariate for resigned acceptance (F_(1106)_ = 5.54, *p* = 0.02, *ηp*^2^ = 0.05) and alternative rewards (F_(1106)_ = 7.19, *p* = 0.01, *ηp*^2^ = 0.06) strategies (see Table 5).

### 3.6. Differences in Coping Strategies Based on Disease Duration (<5 Years; ≥5 Years)

Considering disease duration, groups differed in terms of EDSS: the “longer disease duration” group had a higher EDSS score than the “shorter disease duration” group. In terms of coping strategies, no differences between groups emerged even after controlling for EDSS. As a covariate, EDSS emerged as significant for positive reappraisal (F_(1106)_ = 14.59, *p* < 0.001, *ηp*^2^ = 0.13), resigned acceptance (F_(1106)_ = 8.02, *p* = 0.01, *ηp*^2^ = 0.07), and emotional discharge (F_(1106)_ = 7.75, *p* = 0.01, *ηp*^2^ = 0.07) coping strategies (see Table 6).

## 4. Discussion

The results of the present study showed that coping strategies employed to cope with MS diagnosis played different (positive or negative) roles on current HRQoL. In accordance with previous studies [7,14,34], we found that engagement in approach coping strategies at the time of diagnosis plays a positive role in mental HRQoL of pwMS. We found that strategies aimed at facing MS-related stressors in practical ways, such as problem-solving strategies (i.e., approach/behavioural coping strategies), positively impacted the mental HRQoL of pwMS, even after controlling for disease duration, physical disability, and general distress. In addition, approach coping strategies employed at the time of diagnosis also might have played a positive role in the physical disability of pwMS, after having controlled for disease duration and general distress. Specifically, cognitive strategies aimed at identifying positive aspects of a stressful situation (i.e., personal growth/positive reappraisal strategies) might play a positive role in the physical disability of pwMS. This finding indicates that identifying positive aspects of the situation at the time of diagnosis might predict a better medium/long term physical disability. These results are supported by the higher employment of positive reappraisal coping strategies in patients with normal status/minimal physical disability than in patients with moderate/severe physical disability, in accordance with previous studies (i.e., [22,23,27,28,45]. Taken together, the results of the current study showed that both cognitive and behavioral approach coping strategies employed at the time of diagnosis might have a positive impact on medium/long term mental HRQoL and physical disability of pwMS, even after controlling for disease duration, physical disability, and general distress. Behavioral approach strategies seem to impact mental HRQoL, whereas cognitive approach strategies might play a role in physical disability of pwMS. A possible explanation for these findings is that the engagement in approach coping strategies in general (both cognitive and behavioral) may positively impact mood and, in turn, mental HRQoL and physical disability. Indeed, approach coping strategies imply an active role of patients in managing their illness and related stressors. However, it is worthy of note that the disability status of pwMS might influence the coping style, and reciprocal influence between coping strategies and physical disability might exist (e.g., approach coping strategies contribute to better adherence and, therefore, more effective treatment, which prevents accumulating disability); however, we did not test these reciprocal influences in the current study. Therefore, future studies would benefit from assessing the role played by physical disability in influencing coping strategies and reciprocal influence between coping strategies and physical disability.

On the other hand, in accordance with previous studies [18,34], both cognitive and behavioral *avoidance coping strategies* employed at the time of diagnosis emerged to affect physical and mental HRQoL negatively. Resigned acceptance and emotional discharge employed at the time of diagnosis had the most significant negative impact on HRQoL of pwMS. Both these strategies are usually employed by people when they feel powerless to change, such as when they face chronic illness [8]. Therefore, we may hypothesize that if pwMS at the time of diagnosis perceive little or no control over their chronic illness, they may not engage in appropriate behaviors to promote their own health and, therefore, this may reduce their future HRQoL [27]. In addition, consistent with previous studies (i.e., [22,23,27,28,45]), our findings showed that patients who currently have a moderate/severe physical disability (EDSS > 2) employed greater emotional discharge strategies at the time of diagnosis than patients with a current lower physical disability. Emotional discharge emerged, indeed, as a very problematic coping strategy for pwMS, as previously reported [34]. It is possible that the use of an emotional discharge coping strategy, only aimed at controlling negative emotions associated with the new diagnosis of a chronic illness, may interfere with the engagement in active behaviors to promote the physical health of pwMS.

Interestingly, seeking alternative rewards (i.e., avoidance/behavioural coping strategies) at the time of diagnosis emerged to be positively associated with physical HRQoL. However, this finding is not surprising given that the Alternative Rewards subscale of the self-report questionnaire we employed (i.e., CRI-Adult) only includes adaptive rewards such as engaging in recreational activities that may potentially improve HRQoL of pwMS. Even though seeking alternative rewards may be considered a kind of behavioral disengagement, it might help pwMS to focus on different activities other than their recent diagnosis of MS and physical difficulties. In accordance, previous studies have found that engaging in active lifestyle activities was associated with positive adjustment to MS [46]. More engagement in physical activity seems to be related to fewer reports of fatigue, sleep disturbances, and pain, while social and intellectual activities engagement was associated with fewer depressive symptoms and perceived stress [46].

Finally, we found correlations among avoidance coping strategies, HRQoL, physical disability, and general distress (i.e., the total score of the DASS-21, assessing depression, anxiety, and stress). These findings are in accordance with previous studies showing the negative impact of general distress in pwMS’ HRQoL, physical disability, and coping styles [47,48].

Results of the current study have relevant clinical implications. These findings provide new insights into coping strategies that could be promoted since the time of diagnosis in pwMS. Based on the current findings, pwMS would benefit from early coping-based interventions to enhance positive reappraisal and problem-solving strategies because such strategies have shown to be positively associated with physical disability and mental HRQoL. On the other hand, avoidance coping strategies such as emotional discharge and resigned acceptance should be minimized in pwMS, because these strategies have been linked to poor HRQoL and physical disability. Lastly, seeking positive alternative rewards strategies (such as new activities) at the time of diagnosis should be enhanced in pwMS. These results are important, especially considering that coping strategies are potentially modifiable through psychological interventions and could be taught to pwMS.

The current study is not free from limitations. Firstly, the cross-sectional nature of the study precludes making causal inferences for the relationships we found. Although we have hypothesized that coping strategies might predict both HRQoL and physical disability, these two variables may predetermine a person’s coping strategy choice. Therefore, longitudinal studies should be employed to deepen the causal relationship between coping strategies, HRQoL, and physical disability of pwMS. At the same time, assessing coping strategies in individuals at-risk of developing MS would allow assessing coping strategies before the onset of MS. Secondly, we asked patients to recall the coping strategies they have employed at the time of diagnosis. We investigated their impact on current HRQoL and physical disability; therefore, factors other than coping strategies may impact such variables. Furthermore, the longer the recall period, the less accurate maybe the memory of respondents. However, the diagnosis of MS is a significant event, causing strong emotions. Strong emotions turn an experience into a long-term memory: the stronger the emotion, the longer-lasting the memory [49]. Indeed, people remember facts that are important to their lives. Furthermore, patients of the current study were not affected by severe cognitive impairment, which was an exclusion criterion of the study. Therefore, we believe that our data pertaining to the recall of the diagnosis of MS are reliable.

Despite such limitations, to the best of our knowledge, this is the first study that investigated coping strategies considering MS diagnosis as the core stressor and considering both the focus (approach vs. avoidance) and the method (cognitive vs. behavioral) of coping, and their impact on physical disability and HRQoL of pwMS. Furthermore, to better understand the relationship between coping strategies, HRQoL, and physical disability of pwMS, we have controlled for clinical variables (such as disease duration and EDSS) and general distress: these variables emerged to be prevalent in pwMS and can interfere with HRQoL and physical disability of such patients [47,48]. Notably, disease duration was not significant and did not account for any findings in the present study. Therefore, our results show that coping strategies play a role on HRQoL and physical disability above and beyond the conventional clinical parameters.

These findings support the importance of coping-based interventions from the very beginning of the disease for pwMS (i.e., since the time of diagnosis) with the aim of increasing *adaptive coping strategies* and decreasing *maladaptive coping strategies*, especially given that coping is amenable to change, suggesting that coping strategies should be carefully considered in pwMS since the time of diagnosis to encourage their active disease adjustment to MS disease and promote better clinical outcomes.

## Figures and Tables

**Table 1:** Pearson’s correlations between CRI-Adult subscales, MSQoL-29 physical and mental health composite scores, EDSS, and disease duration.

	1	2	3	4	5	6	7	8	9	10	11	12	13
**1.** CRI-Adult Logical Analysis	1												
**2.** CRI-Adult Positive Reappraisal	0.47 **	1											
**3.** CRI-Adult Support Seeking	0.48 **	0.45 **	1										
**4.** CRI-Adult Problem Solving	0.58 **	0.45 **	0.54 **	1									
**5.** CRI-Adult Cognitive Avoidance	0.01	0.01	−0.12	−0.15	1								
**6.** CRI-Adult Resigned Acceptance	0.30 **	0.05	0.22 *	0.03	0.40 **	1							
**7.** CRI-Adult Alternative Rewards	0.48 **	0.51 **	0.45 **	0.54 **	−0.05	0.09	1						
**8.** CRI-Adult Emotional Discharge	0.32 **	0.12	0.18	0.05	0.44 **	0.41 **	0.29 **	1					
**9.** MSQOL-29 Physical Health	−0.04	0.13	−0.04	0.24 *	−0.24 *	−0.40 **	0.25 **	−0.28 **	1				
**10.** MSQOL-29 Mental Health	−0.12	0.01	−0.11	0.21 *	−0.31 **	−0.41 **	0.09	−0.46 **	0.75 **	1			
**11.** EDSS	−0.05	−0.35 **	0.08	−0.07	−0.06	0.24 *	−0.02	0.29 **	−0.54 **	−0.39 **	1		
**12.** Disease Duration	−0.08	−0.02	−0.07	0.05	−0.18	−0.20 *	0.24 *	0.14	0.08	0.11	0.20 *	1	
**13.** DASS-21 total score	0.04	0.03	0.05	−0.18	0.37 **	0.26 **	0.02	0.46 **	−0.38 **	−0.56 **	0.28 **	−0.04	1

*Note.* * *p* < 0.05; ** *p* < 0.001; CRI-Adult: Coping Responses Inventory-Adult Form; MSQoL-29: Multiple Sclerosis Quality of Life-29; EDSS: Expanded Disability Status Scale; DASS-21: Depression Anxiety Stress Scale-21.

**Table 2:** Results of the multiple regression analysis predicting MSQoL-29 physical health composite score.

Predictors	B	ES	β	t	ΔR2	F	gdl
Step 1					*p* < 0.001	20.65	3102
Constant	77.68	2.82		27.55			
Disease duration	0.50	0.21	0.18	2.32 *			
EDSS	−6.05	0.98	−0.51	−6.14 ***			
DASS-21 total score	−0.30	0.14	−0.23	−2.77 **			
Step 2					*p* < 0.001	12.59	897
Constant	81.32	6.99		11.63			
Disease duration	0.17	0.21	0.06	0.78			
EDSS	−5.32	0.97	−0.45	−5.49 ***			
DASS-21 total score	−0.20	0.14	−0.12	−1.41			
CRI-Adult Problem Solving	0.19	0.46	0.04	0.42			
CRI-Adult Cognitive Avoidance	−0.49	0.44	−0.09	−1.09			
CRI-Adult Resigned Acceptance	−1.27	0.44	−0.25	−2.91 **			
CRI-Adult Alternative Rewards	1.05	0.40	0.24	0.24 *			
CRI-Adult Emotional Discharge	−0.27	0.48	−0.05	−0.05			

Note. * *p* < 0.05; ** *p* < 0.01; *** *p* < 0.001; EDSS: Expanded Disability Status Scale; DASS-21: Depression Anxiety Stress Scale-21; CRI-Adult: Coping Responses Inventory-Adult Form.

**Table 3:** Results of the multiple regression analysis predicting MSQoL-29 mental health composite score.

Predictors	B	ES	β	t	ΔR2	F	gdl
Step 1					*p* < 0.001	21.99	3102
Constant	79.20	2.68		29.50			
Disease duration	0.37	0.20	0.14	1.80			
EDSS	−3.18	0.94	−0.28	−3.38 ***			
DASS-21 total score	−0.78	0.13	−0.48	−5.94 ***			
Step 2					*p* < 0.001	12.88	798
Constant	79.06	6.91		11.44			
Disease duration	0.33	0.21	0.13	1.62			
EDSS	−2.46	0.94	−0.21	−2.60 *			
DASS-21 total score	−0.54	0.14	−0.33	−3.80 ***			
CRI-Adult Problem Solving	0.81	0.38	0.16	2.13 *			
CRI-Adult Cognitive Avoidance	0.04	0.44	0.01	0.08			
CRI-Adult Resigned Acceptance	−0.80	0.43	−0.16	−1.87			
CRI-Adult Emotional Discharge	−0.99	0.46	−0.21	−2.15 *			

*Note.* * *p* < 0.05; *** *p* < 0.001; EDSS: Expanded Disability Status Scale; DASS-21: Depression Anxiety Stress Scale-21; CRI-Adult: Coping Responses Inventory-Adult Form.

**Table 4:** Results of multiple regression analysis predicting EDSS.

Predictors	B	ES	β	t	ΔR2	F	gdl
Step 1					*p* = 0.001	7.04	2103
Constant	0.75	0.27		2.75			
Disease duration	0.05	0.02	0.21	2.23 *			
DASS-21 total score	0.04	0.01	0.29	3.10 **			
Step 2					*p* < 0.001	8.15	5100
Constant	2.19	0.67		3.28			
Disease duration	0.05	0.02	0.20	2.29 *			
DASS-21 total score	0.03	0.01	0.19	2.00 *			
CRI-Adult Positive Reappraisal	−0.18	0.04	−0.36	−4.25 ***			
CRI-Adult Resigned Acceptance	0.08	0.04	0.17	1.83			
CRI-Adult Emotional Discharge	0.05	0.04	0.13	1.22			

*Note.* * *p* < 0.05; ** *p* < 0.01; *** *p* < 0.001; EDSS: Expanded Disability Status Scale; DASS-21: Depression Anxiety Stress Scale-21; CRI-Adult: Coping Responses Inventory-Adult Form.

**Table 5:** Differences in demographic data, disease duration, and coping strategies between the “normal/minimal physical disability” and the “moderate/severe physical disability” groups.

	Normal/Minimal Physical Disability Group (*N* = 63) M (SD)	Moderate/Severe Disability Group (*N* = 45) M (SD)	t_(106)_/F_(1106)_	*p*	*ηp* ^2^
Age	36.92 (10.45)	39.73 (10.80)	−1.36	0.18	-
Education	14.29 (3.06)	14.33 (3.51)	−0.07	0.94	-
Disease duration	5.21 (6.11)	7.96 (7.05)	−2.16	0.03	0.04
CRI-Adult Logical Analysis	9.84 (3.52)	9.64 (3.64)	0.01	0.91	-
CRI-Adult Positive Reappraisal	13.17 (2.68)	11.71 (3.06)	6.89	0.01	0.06
CRI-Adult Support Seeking	11.59 (2.99)	12.33 (2.77)	2.25	0.14	-
CRI-Adult Problem Solving	12.43 (3.28)	12.22 (3.67)	0.17	0.68	-
CRI-Adult Cognitive Avoidance	9.92 (3.70)	9.13 (3.47)	0.58	0.45	-
CRI-Adult Resigned Acceptance	8.81 (3.46)	9.56 (3.53)	2.49	0.12	-
CRI-Adult Alternative Rewards	10.22 (4.11)	9.89 (4.05)	0.94	0.33	
CRI-Adult Emotional Discharge	6.62 (3.50)	8.33 (3.46)	5.10	0.03	0.05

*Note.* CRI-Adult: Coping Responses Inventory-Adult Form.

**Table 6:** Differences between groups (less and more than five years) in coping strategies based on disease duration.

	<5 Years Group (*N* = 61) M (SD)	≥5 Years Group (*N* = 47) M (SD)	t_(106)_/F_(1106)_	*p*
EDSS	1.36 (1.25)	2 (1.75)	−2.19	0.03
CRI-Adult Logical Analysis	9.90 (3.71)	9.29 (3.19)	0.61	0.44
CRI-Adult Positive Reappraisal	12.54 (3.15)	12.42 (2.61)	0.35	0.55
CRI-Adult Support Seeking	12.03 (2.91)	11.53 (2.70)	1.21	0.27
CRI-Adult Problem Solving	12.17 (3.37)	12.38 (3.48)	0.22	0.64
CRI-Adult Cognitive Avoidance	9.46 (3.19)	8.62 (3.64)	0.63	0.43
CRI-Adult Resigned Acceptance	8.81 (3.46)	9.56 (3.53)	3.45	0.07
CRI-Adult Alternative Rewards	9.39 (3.98)	10.73 (4.09)	3.09	0.08
CRI-Adult Emotional Discharge	6.71 (3.35)	7.87 (3.68)	1.19	0.28

*Note.* CRI-Adult: Coping Responses Inventory-Adult Form.

## Data Availability

The data that support the findings of this study are available from the last author, M.C., upon reasonable request.

## Appendix A

Means and standard deviations obtained by pwMS on CRI-Adult, MSQoL-29, and DASS-21.

**Table A1:** CRI-Adult scores obtained by patients with MS.

CRI-Adult Subscales	CRI-Adult Scores M (SD)
CRI-Adult Logical Analysis	9.76 (3.55)
CRI-Adult Positive Reappraisal	12.56 (2.92)
CRI-Adult Support Seeking	11.90 (2.91)
CRI-Adult Problem Solving	12.34 (3.43)
CRI-Adult Cognitive Avoidance	9.59 (3.61)
CRI-Adult Resigned Acceptance	9.12 (3.49)
CRI-Adult Alternative Rewards	10.08 (4.07)
CRI-Adult Emotional Discharge	7.33 (3.57)

*Note.* CRI-Adult: Coping Responses Inventory-Adult Form.

**Table A2:** MSQoL-29 scores obtained by patients with MS.

MSQoL-29 Subscales	MSQoL-29 Scores M (SD)
Physical function	80.71 (25.92)
Bodily pain	75.65 (21.63)
Emotional well-being	64.54 (19.02)
Energy	50.91 (21.12)
Cognitive function	67.50 (20.92)
Health distress	74.85 (21.25)
Sexual function	78.96 (30.76)
Social function (single item)	65.14 (26.62)
Health perception (single item)	52.94 (27.24)
Change in health (single item)	46.76 (27.79)
Overall quality of life (single item)	66.57 (20.70)
Physical Health Composite	65.89 (17.75)
Mental Health Composite	65.23 (17.33)

*Note.* MSQoL-29: Multiple Sclerosis Quality of Life-29.

**Table A3:** DASS-21 scores obtained by patients with MS.

DASS-21 Subscales and Total Score	DASS-21 Scores M (SD)
DASS-21-Depression	3.90 (4.19)
DASS-21-Anxiety	3 (3.20)
DASS-21-Stress	7.25 (4.61)
DASS-21 Total score	14.15 (10.61)

*Note.* DASS-21: Depression Anxiety Stress Scale-21.

## References

[1] Lassmann H. (2010). Axonal and Neuronal Pathology in Multiple Sclerosis: What Have We Learnt from Animal Models. Exp. Neurol..

[2] Oh J., Vidal-Jordana A., Montalban X. (2018). Multiple Sclerosis: Clinical Aspects. Curr. Opin. Neurol..

[3] Grzegorski T., Losy J. (2017). Cognitive Impairment in Multiple Sclerosis—A Review of Current Knowledge and Recent Research. Rev. Neurosci..

[4] Glanz B.I., Dégano I.R., Rintell D.J., Chitnis T., Weiner H.L., Healy B.C. (2012). Work Productivity in Relapsing Multiple Sclerosis: Associations with Disability, Depression, Fatigue, Anxiety, Cognition, and Health-Related Quality of Life. Value Health.

[5] Nogueira L.A.C., Nóbrega F.R., Lopes K.N., Thuler L.C.S., Alvarenga R.M.P. (2009). The Effect of Functional Limitations and Fatigue on the Quality of Life in People with Multiple Sclerosis. Arq. Neuro-Psiquiatr..

[6] Benedict R.H.B., Wahlig E., Bakshi R., Fishman I., Munschauer F., Zivadinov R., Weinstock-Guttman B. (2005). Predicting Quality of Life in Multiple Sclerosis: Accounting for Physical Disability, Fatigue, Cognition, Mood Disorder, Personality, and Behavior Change. J. Neurol. Sci..

[7] Goretti B., Portaccio E., Zipoli V., Hakiki B., Siracusa G., Sorbi S., Amato M.P. (2010). Impact of Cognitive Impairment on Coping Strategies in Multiple Sclerosis. Clin. Neurol. Neurosurg..

[8] Lazarus R., Folkman S. (1984). Stress, Appraisal, and Coping.

[9] Carver C.S., Scheier M.F. (1994). Situational Coping and Coping Dispositions in a Stressful Transaction. J. Personal. Soc. Psychol..

[10] Carver C.S., Scheier M.F., Weintraub K.J. (1989). Assessing Coping Strategies: A Theoretically Based Approach. J. Personal. Soc. Psychol..

[11] Penley J.A., Tomaka J., Wiebe J.S. (2002). The Association of Coping to Physical and Psychological Health Outcomes: A Meta-Analytic Review. J. Behav. Med..

[12] Suls J., Fletcher B. (1985). The Relative Efficacy of Avoidant and Nonavoidant Coping Strategies: A Meta-Analysis. Am. Psychol. Assoc..

[13] Aldao A., Nolen-Hoeksema S., Schweizer S. (2010). Emotion-Regulation Strategies across Psychopathology: A Meta-Analytic Review. Clin. Psychol. Rev..

[14] McCabe M. (2006). A Longitudinal Study of Coping Strategies and Quality of Life among People with Multiple Sclerosis. J. Clin. Psychol. Med. Settings.

[15] Keramat Kar M., Whitehead L., Smith C.M. (2019). Characteristics and Correlates of Coping with Multiple Sclerosis: A Systematic Review. Disabil. Rehabil..

[16] Dempster M., Howell D., McCorry N.K. (2015). Illness Perceptions and Coping in Physical Health Conditions: A Meta-Analysis. J. Psychosom. Res..

[17] Devy R., Lehert P., Varlan E., Genty M., Edan G. (2015). Improving the Quality of Life of Multiple Sclerosis Patients through Coping Strategies in Routine Medical Practice. Neurol. Sci..

[18] McCabe M.P., McKern S., McDonald E. (2004). Coping and Psychological Adjustment among People with Multiple Sclerosis. J. Psychosom. Res..

[19] Montel S.R., Bungener C. (2007). Coping and Quality of Life in One Hundred and Thirty Five Subjects with Multiple Sclerosis. Mult. Scler. J..

[20] Pakenham K.I., Stewart C.A., Rogers A. (1997). The Role of Coping in Adjustment to Multiple Sclerosis-Related Adaptive Demands. Psychol. Health Med..

[21] Hanna M., Strober L.B. (2020). Anxiety and Depression in Multiple Sclerosis (MS): Antecedents, Consequences, and Differential Impact on Well-Being and Quality of Life. Mult. Scler. Relat. Disord..

[22] Lode K., Bru E., Klevan G., Myhr K.M., Nyland H., Larsen J.P. (2010). Coping with Multiple Sclerosis: A 5-Year Follow-up Study. Acta Neurol. Scand..

[23] Milanlioglu A., Özdemir P.G., Cilingir V., Gülec T.Ç., Aydin M.N., Tombul T. (2014). Coping Strategies and Mood Profiles in Patients with Multiple Sclerosis. Arq. Neuro-Psiquiatr..

[24] Santangelo G., Corte M.D., Sparaco M., Miele G., Garramone F., Cropano M., Esposito S., Lavorgna L., Gallo A., Tedeschi G. (2021). Coping strategies in relapsing–remitting multiple sclerosis non-depressed patients and their associations with disease activity. Acta Neurol. Belg..

[25] Wilski M., Gabryelski J., Brola W., Tomasz T. (2019). Health-related quality of life in multiple sclerosis: Links to acceptance, coping strategies and disease severity. Disabil Health J..

[26] Kurtzke J.F. (1983). Rating Neurologic Impairment in Multiple Sclerosis: An Expanded Disability Status Scale (EDSS). Neurology.

[27] Holland D.P., Schlüter D.K., Young C.A., Mills R.J., Rog D.J., Ford H.L., Orchard K. (2019). Use of Coping Strategies in Multiple Sclerosis: Association with Demographic and Disease-Related Characteristics ✰. Mult. Scler. Relat. Disord..

[28] Lorefice L., Fenu G., Frau J., Coghe G., Marrosu M.G., Cocco E. (2018). The Burden of Multiple Sclerosis and Patients’ Coping Strategies. BMJ Supportive Palliat. Care.

[29] Beatty W.W., Hames K.A., Blanco C.R., Williamson S.J., Wilbanks S.L., Olson K.A. (1998). Correlates of Coping Style in Patients with Multiple Sclerosis. Mult. Scler..

[30] Haase C.G., Lienemann M., Faustmann P.M. (2007). Neuropsychological Deficits but Not Coping Strategies Are Related to Physical Disability in Multiple Sclerosis. Eur. Arch. Psychiatry Clin. Neurosci..

[31] Rätsep T., Kallasmaa T., Pulver A., Gross-Paju K. (2000). Personality as a Predictor of Coping Efforts in Patients with Multiple Sclerosis. Mult. Scler. (Houndmills Basingstoke Engl.).

[32] Aldwin C.M., Park C.L. (2004). Coping and Physical Health Outcomes: An Overview. Psychol. Health.

[33] Moos R. (1993). Coping Responses Inventory. CRI-Adult Form.

[34] Grech L.B., Kiropoulos L.A., Kirby K.M., Butler E., Paine M., Hester R. (2018). Target Coping Strategies for Interventions Aimed at Maximizing Psychosocial Adjustment in People with Multiple Sclerosis. Int. J. MS Care.

[35] Goretti B., Portaccio E., Zipoli V., Razzolini L., Amato M.P. (2010). Coping Strategies, Cognitive Impairment, Psychological Variables and Their Relationship with Quality of Life in Multiple Sclerosis. Neurol. Sci..

[36] McDonald W.I., Compston A., Edan G., Goodkin D., Hartung H.P., Lublin F.D., McFarland H.F., Paty D.W., Polman C.H., Reingold S.C. (2001). Recommended Diagnostic Criteria for Multiple Sclerosis: Guidelines from the International Panel on the Diagnosis of Multiple Sclerosis. Ann. Neurol..

[37] Pitteri M., Romualdi C., Magliozzi R., Monaco S., Calabrese M. (2017). Cognitive impairment predicts disability progression and cortical thinning in MS: An 8-year study. Mult. Scler..

[38] Scozzari S., di Pietro M., Ghisi M. (2015). Coping Responses Inventory.

[39] Rosato R., Testa S., Bertolotto A., Confalonieri P., Patti F., Lugaresi A., Grasso M.G., Toscano A., Giordano A., Solari A. (2016). Development of a Short Version of MSQOL-54 Using Factor Analysis and Item Response Theory. PLoS ONE.

[40] Lovibond P.F., Lovibond S.H. (1995). The Structure of Negative Emotional States: Comparison of the Depression Anxiety Stress Scales (DASS) with the Beck Depression and Anxiety Inventories. Behav. Res. Ther..

[41] Bottesi G., Ghisi M., Altoè G., Conforti E., Melli G., Sica C. (2015). The Italian Version of the Depression Anxiety Stress Scales-21: Factor Structure and Psychometric Properties on Community and Clinical Samples. Compr. Psychiatry.

[42] Rezapour A., Almasian Kia A., Goodarzi S., Hasoumi M., Nouraei Motlagh S., Vahedi S. (2017). The Impact of Disease Characteristics on Multiple Sclerosis Patients’ Quality of Life. Epidemiol. Health.

[43] Gulick E.E. (1994). Social Support among Persons with Multiple Sclerosis. Res. Nurs. Health.

[44] Strober L.B. (2018). Quality of Life and Psychological Well-Being in the Early Stages of Multiple Sclerosis (MS): Importance of Adopting a Biopsychosocial Model. Disabil. Health J..

[45] Mohr D.C., Goodkin D.E., Nelson S., Cox D., Weiner M. (2002). Moderating Effects of Coping on the Relationship between Stress and the Development of New Brain Lesions in Multiple Sclerosis. Psychosom. Med..

[46] Strober L.B., Becker A., Randolph J.J. (2018). Role of Positive Lifestyle Activities on Mood, Cognition, Well-Being, and Disease Characteristics in Multiple Sclerosis. Appl. Neuropsychol. Adult.

[47] Fruewald S., Loeffler-Stastka H., Eher R., Saletu B., Baumhacki U. (2001). Depression and Quality of Life in Multiple Sclerosis. Acta Neurol. Scand..

[48] Amato M.P., Ponziani G., Rossi F., Liedl C.L., Stefanile C., Rossi L. (2001). Quality of Life in Multiple Sclerosis: The Impact of Depression, Fatigue and Disability. Mult. Scler. (Houndmills Basingstoke Engl.).

[49] Alberini C.M. (2010). Long-term Memories: The Good, the Bad, and the Ugly. Cerebrum.

